# The complete mitochondrial genome of *Sanghuangporus vaninii* Zhehuang-1 (Hymenochaetales, Basidiomycota)

**DOI:** 10.1080/23802359.2020.1832592

**Published:** 2021-03-19

**Authors:** Tingting Song, Fei Xu, Yingyue Shen, Lijun Fan, Weiming Cai

**Affiliations:** a Institute of Horticulture, Zhejiang Academy of Agricultural Sciences, Hangzhou, Zhejiang, People’s Republic of China; b Central Laboratory, Zhejiang Academy of Agricultural Sciences, Hangzhou, Zhejiang, People’s Republic of China

**Keywords:** *Sanghuangporus vaninii*, *Inonotus linteus* complex, mitochondrial genome, phylogenetic analysis

## Abstract

In this study, the complete mitochondrial genome of *Sanghuangporus vaninii* Zhehuang-1 was determined. Genomic DNA samples individually collected from a population in southeastern China (Qiandaohu, Zhejiang Province, N29°44′24′′, E118°52′48′′) were sequenced with an Illumina NovaSeq 6000. The complete mitochondrial genome was 97,345 bp in length and consisted of 22 protein-coding genes (PCGs), 29 tRNAs, 3 ribosomal RNAs, and a control region. *Sanghuangporus vaninii* has a mitochondrial gene arrangement that is similar to that of *S. sanghuang*. Phylogenetic analysis performed using ML methods based on the complete mitogenome sequence showed that *S. vaninii* is a member of the Polyporaceae. The complete mitogenome sequence provides important data for further study of the *Inonotus linteus* complex.

The genus *Sanghuangporus* is one of the most popular medicinal polypore mushroom groups in east Asia. Their basidiocarps are perennial and their appearance is effused-reflexed to pileate. The surface of the pileus is brown to black with a radially cracked crust. The pore surface color is yellow to brown. Their hyphal system is heterogeneous with a monomitic context and a dimitic hymenophoral trama. Their basidiospores are broadly ellipsoid to subglobose, yellowish, and thick-walled (Zhou et al. 2016). The genus is widely distributed in boreal forests which are found in subtropical to tropical zones in China. They grow on angiosperm wood, causing a white rot (Tian et al. 2013). For the past several decades, researchers have mainly focused on the pharmacology and taxonomy of these species, so data on only one mitogenome was available (Han et al. 2018). Here, we sequenced the complete mitochondrial genome of *Sanghuangporus vaninii* Zhehuang-1. The complete mitogenome data will contribute to constructing a more comprehensive understanding and the further study of the *Inonotus linteus* complex.

Specimens of *S. vaninii* were collected from the mushroom farm of the Sangdu Cooperative in Qiandaohu (N29°44′24′′, E118°52′48′′), Hangzhou, Zhejiang Province, and the strain Zhehuang-1 was isolated from the basidiocarps grown in culture substrates. It was identified as *S. vaninii* based on microscopic characteristics and rRNA gene sequencing by the Institute of Microbiology, Chinese Academy of Sciences (Beijing, China). The whole mitochondrial genome was sequenced with an Illumina NovaSeq 6000 (Illumina, San Diego, CA) and assembled using the CLC Protein Workbench. Coding genes were predicted using fgenesB (Solovyev and Salamov 2011). The genes were blasted against the NR database (cut-off e-value of 1e^−5^). Other genomic elements were annotated using the online MITOS server (Bernt et al. 2013).

The complete mitogenome of *S. vaninii* Zhehuang-1 is 97,345 bp in length (Genbank accession number: MT227806), and a double-stranded molecular weight of 60.123 MDa. The mitochondria contained 54 genes which consisted of 22 PCGs (4 were hypothetical), 29 transfer RNA genes (tRNAs), 3 ribosomal RNA genes (rRNAs), and a control region. The overall base composition was 37.7% A, 38.9% T, 10.9% G, and 12.6% C with an obvious A＋T bias of 76.5%. Both the GC-skew (–0.074) and AT-skew (–0.015) for the whole mitogenome were slightly negative, indicating a higher occurrence of Cs than Gs and Ts than As. One of the PCGs (nad5) used TTG and one PCG (nad1) used GTG as the initiation codon, while the other 20 PCGs used the typical ATG start codon. The lengths of the tRNAs ranged from 53 to 87 bp and the rRNA exons ranged from 91 to 1640 bp.

To ascertain the phylogenetic position of *S. vaninii*, 13 other mitochondrial genome sequences which belong to 9 Polyporales species, two other mushroom species, one Ascomycota, and the model plant *Arabidopsis Thaliana* were aligned in MAFFT version 7.471 (Katoh and Standley 2013) and then trimmed using trimAL version 1.4.rev15 (the ‘automated1’ option) (Capella-Gutiérrez et al. 2009). The maximum likelihood (ML) analysis with the combined rapid bootstrap (1000 replicates) was conducted using RAxML version 8.2.12 and the search for the ML tree was conducted with the options ‘-f a’(Stamatakis 2014). The ML tree used the GTRGAMMA model for analysis. The phylogenetic tree showed that *S. vaninii* was sister to *S. sanghuang* (Figure 1).

**Figure 1.**
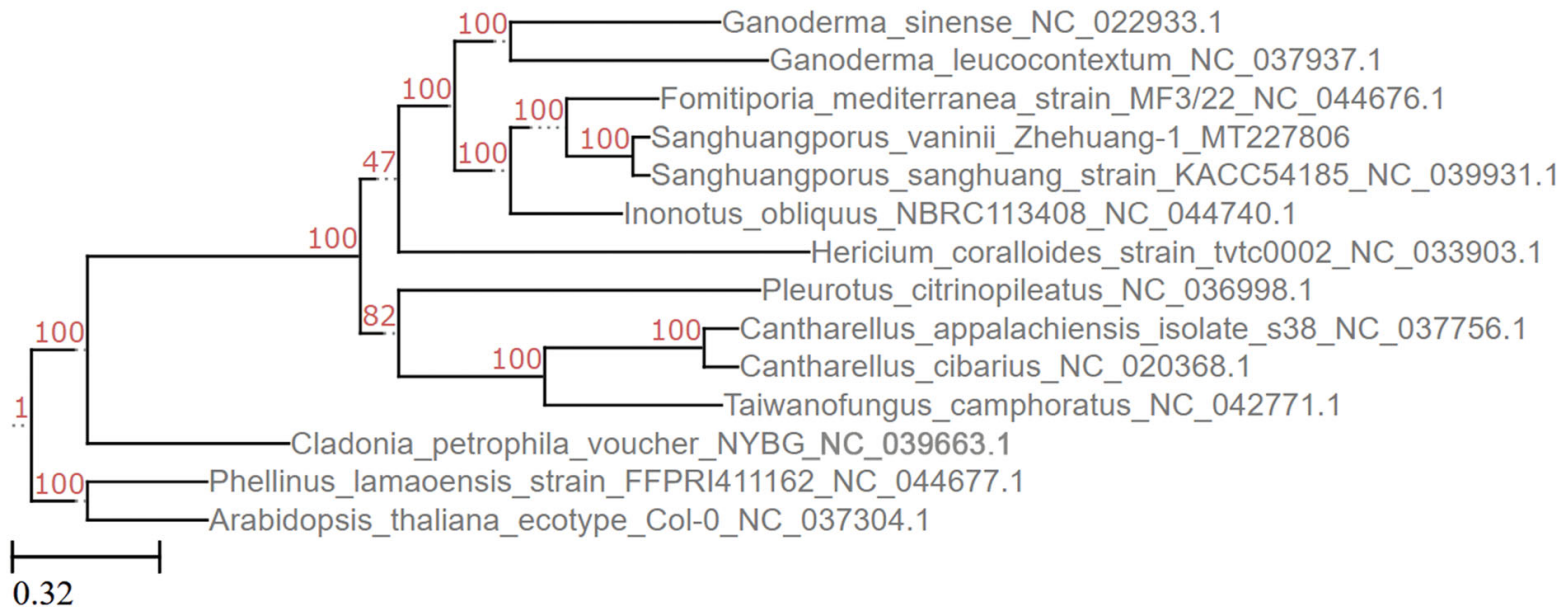
Phylogenetic position of *S. vaninii* by the ML method based on the mitochondrial genome sequences from 14 species as outgroups. Values along branches correspond to ML bootstrap percentages.

## Data Availability

The data that support the findings of this study are openly available in the NCBI Genbank （accession number: MT227806） at https://www.ncbi.nlm.nih.gov/genbank/.
